# Research and Remedies

**Published:** 1912-03-16

**Authors:** 


### RESEARCH AND REMEDIES.

### The Meiostagmine Reaction.

This serological test, which was described by Ascoli and his pupils in 1910, has been extensively employed in Italy by numerous investigators as a diagnostic aid in all sorts and conditions of disease. The principles upon which it is based are extremely complex, but, according to the Italian authors, it can be used for the diagnosis of such diverse conditions as typhoid fever, syphilis, malignant disease, ankylostomiasis, and others. The test does not, it was said, differentiate sarcoma from carcinoma, but affords a reliable indication between innocency and malignancy of tumours. It is curious that outside Italy practically no one is able to confirm the findings of Ascoli and his school; in Germany, at Bier's clinic, they even went to the length of importing Italian antigen in the endeavour to make the test work, but all in vain. In the *American Journal of the Medical Sciences* Bernstein and Simons report a similar sequence of unbroken failure. They adopted in turn all the modifications published from time to time by the Italians, but neither with the original nor any subsequent technique could they get any satisfactory results. It was not that the test gave results which were out of harmony with the clinical and microscopical evidence; but simply that they could not get the "meiostagmine reaction" to come off at all. Thus in typhoid fever, syphilis, and malignant disease they failed to get anything that could be called a positive reaction even when the diagnoses made in other ways (and also post-mortem in some cases) were unassailable. The paper in which these results are published gives a full bibliography up to the early part of 1911, and it is certainly strange that with such a chorus of enthusiastic approval from the Italian literature, the verdict of everyone else is so hostile. It may be added that Bernstein and Simons advance some pertinent criticisms of the test, both as to principles and as to the many modifications of technique which have been published.

### The Actions of the Psoas Muscle.

The effects of muscular action in cases of fracture, dislocation, and deformities of various kinds, are admitted to be highly important; but very little first-hand information is available about the exact actions of individual muscles in specified cases. All that most surgical authors will commit themselves to are statements copied from anatomical textbooks, often given with some reserve. It is therefore of interest to note that two surgeons have recently (and independently) been investigating the exact functions of the ilio-psoas muscle and its influence in various morbid lesions of the thigh and hip. Mr. MacLennan published his views last year in the *British Medical Journal*, and Mr. Buckley now contributes his to the *Medical Chronicle*. The latter is substantially in agreement with the former; he regards the muscle as an internal rotator under normal conditions, and thinks that it helps to flex the thigh when flexion is already advanced, but cannot commence this action when the limb is extended. After (unimpacted) fracture of the femur above the lesser trochanter, the muscle becomes an external rotator, thus helping to produce the characteristic position of the external rotation. In the second stage of tuberculous disease of the hip-joint, the fact that the strong tendon of the muscle is in contact with the joint, while the muscle belly for the most part is not, renders it possible for extensive destruction to take place among the external rotators at the back of the joint while the ilio-psoas remains practically unaffected; thus the latter is enabled to exert fully its internal rotatory action and bring about the characteristic deformity of this stage. He further shows that at a certain stage in coxa valga the rotatory action changes from internal to external, and the resulting upset of muscular equilibrium produces the external rotation typical of the condition. These conclusions are based upon a very thoughtful analysis of facts disclosed by anatomical study; it is not often, unfortunately, that research in anatomy is able to afford such help to clinical surgery as in this case.

### The Prevention of Enlarged Prostate.

Hitherto surgeons have plumed themselves on the improvements in their art by which the enlarged prostate can now be safely removed. If Doctors Ballenger and Elder are right in the contentions which they have put forward in *International Clinics*, the prevention of prostatic hypertrophy is now a feasible aim; this would be an even greater boon to mankind than the discovery a few years ago of the correct principles of removal of that gland. Their treatment is by means of mixed vaccines, and is based upon the supposition that prostatic hypertrophy is due to the long continued irritation by pervading microbes. Of these microbes the gonococcus is less important, they incline to believe, than some of the pus-forming organisms which so often complicate the ravages of the former. The authors expressly exclude cancer, adenoma, and other growths from the scope of their remarks upon prevention. They also speak very highly of a new method of aborting a very early gonorrhœa; this consists of the injection into the anterior urethra of fifteen to twenty minims of a 5 per cent. solution of argyrol, which is then sealed in with collodion and retained for five or six hours. The bladder must first be emptied, and the fluid intake be restricted so that it may not fill up again too rapidly. This method is only of use when the inflammation is limited to the anterior urethra; but it is said to cure 90 per cent. of such cases. The treatment is repeated every day until cure results, usually after the second, third, or fourth injection. If enlarged prostate really is due to chronic inflammation rather than to adenomatous changes in most cases, this line of prevention may be effective; but the authors have not by any means proved this premiss of their syllogism.

